# Learning from plants: is it possible to make an animal organism a non-host for cancer?

**DOI:** 10.3389/fonc.2026.1781838

**Published:** 2026-04-15

**Authors:** Lev G. Nemchinov

**Affiliations:** U.S. Department of Agriculture, Agricultural Research Service, Beltsville Agricultural Research Center, Beltsville, Molecular Plant Pathology Laboratory, Beltsville, MD, United States

**Keywords:** cancer, cancer immunity, non-cancer environment, non-host resistance, plant immunity

## Abstract

**Background:**

Cancer is one of the most significant global health problems and a leading cause of death worldwide. Plants have a broad-spectrum mechanism of defense against pathogens called non-host resistance (NHR), when an entire plant species is resistant to all isolates of a microbial species. As nearly all basic mechanisms and components of the NHR in plants have similarities to cancer responses in vertebrates, it is theoretically possible to make an animal organism an absolute non-host for cancer by generating a total non-cancer environment, an animal analog of the NHR.

**Methods:**

An integrative review of cancer-causing events and defense mechanisms was conducted drawing parallels with a broad-spectrum immunity against pathogens in plants, known as non-host resistance, where an entire plant species is resistant to all isolates of a microbial species.

**Results:**

Based on the currently available literature, the hypothesis suggests that the fundamental principles and mechanisms underlying NHR in plants might be applicable to animal organisms, potentially enabling the establishment of a hypothetical non-cancer environment characterized by absolute and durable immunity against cancer.

**Conclusions:**

Within the current accumulated knowledge of the processes related to the NHR and to the body’s natural and induced defenses against cancer, this hypothesis appears to be experimentally testable. The testing could be conducted through various strategies, integrating methods used for artificially inducing or engineering the NHR in plants, for example genetically modifying animals to possess traits associated with cancer resistance or enhancing their immune response via a combination of immunotherapies and trained immunity.

## Introduction

Cancer is among the most important health problems and a leading cause of death worldwide. There is no universal cure against this complex disease as it is highly diverse and heterogenous. A typical process of tumorigenesis involves multiple steps, including disrupted cell cycle regulation, mutations in growth factors and signaling pathways, inactivation of tumor suppressor genes, activation of oncogenes, DNA repair deficiencies, mutations in DNA repair genes, evasion of apoptosis, resistance to immune surveillance, limitless replicative potential, and so forth (1, 2).

Each of these and many other reasons for cancer onset are extremely complex, with thousands of possible ways to intervene without assuredly knowing if the intervention will be successful. Ideally, the root causes and processes would have to be eliminated as a whole because they are interconnected, often derive from and initiate one another.

Since cancer-related events occur naturally over time or arise consequentially from anthropogenic activities, the possibility of a simultaneous and total halt to their development appears highly unlikely. A more realistic alternative would be to prevent the product of their combined activities – tumor – from happening, by creating unlivable conditions for the mass, so that “it does not feel like growing” due to the hostile environment. This credible option is, surprisingly, still largely unexplored.

In nature, all species have their unique habitat, a specific ecological niche (3) outside of which they cannot occur. Cancer cells have their niche as well, a specialized microenvironments where they reside hijacking host defenses (4, 5). Although metastases are considered a spread of cancer cells away from their primary niche, similar *favorable* environments are selected and eventually colonized at the new locations (6).

The assumption of no niche for cancer prompts more questions, the most important of them being how to create an all-embracing and hostile for the emergence and proliferation of cancer cells environment within an individual organism. So that if any of the cancer-causing events occur, they will have little or no chance to transform into a tumor.

Plants have a broad-spectrum mechanism of defense against pathogens called non-host resistance (NHR), when an entire plant species is resistant to all isolates of a microbial species (7). For example, immunity of rice against all rust and powdery mildew fungi (8). Or general resistance of monocotyledonous plants against broadly successful in dicots plant pathogen *Agrobacterium tumefaciens*, a cause of the neoplastic disease crown gall (9). While NHR is effective against pathogens and cancer cells are perceived by an organism as “self” or “altered self”, tumors are still de facto “foreign intruders” since they generate neoantigens, novel proteins absent from normal tissues. Considering this along with the fundamental differences in cellular anatomy, pathogen recognition strategies, and tissue mobility, would that be theoretically possible to make an animal organism an absolute non-host for cancer? As nearly all basic mechanisms and components of the NHR in plants have similarities to cancer responses in animals, this challenging task may be more achievable than it first appears.

## Main text

The evolution operates at all levels, from molecular to organismal, obeying the same law of natural selection. In nature, species that confront an unfavorable environment can adapt, enter dormancy, migrate, or face extinction. The ability of cancer cells to undergo the first three states and also to overcome harsh treatment conditions is well known (10, 11). But while cancer cells can survive hostile surroundings, will they, given a choice, likely emerge *de novo* in adverse or in the favorable environment? Surprisingly, the literature on this simple query is not as extensive as it would imply, if any, and mostly relates to the ability of cancer cells to adapt and resist different stress conditions (12).

Furthermore, would cancer cells emerge *at all* if the entire organismal environment is hostile to their appearance and proliferation? Presumably, they would not: while cancer cells can post-factum resist the adverse environment, they are unlikely to arise in it. Reiterating the point, it is about the odds of cancer cells *emerging* in an extremely hostile environment rather than about their ability to adapt and persist within it.

There are only a few comprehensive, all-organismic conditions that can potentially satisfy a requirement of being favorable for normal but hostile for cancer cells. Among them are oxygen levels, aerobic in normal cells and hypoxic in solid tumor environments (13) and nutrient dependencies, particularly glucose availability: while normal cells require a balanced supply of nutrients, cancer competes for nutrients like glucose and glutamine, relying on glycolysis and much higher glycose uptake (14). None of these, however, can prevent cancer cells from appearing: they can emerge and grow in well-oxygenated and survive in limited glycose environment (15, 16). Other known candidates are many but more specific, targeting individual cancer-associated processes rather than being broadly detrimental to all cancer cells.

The NHR is a remarkably durable and long-lasting immunity and the sole reason why most plants in the natural environment are healthy. It is triggered by a combination of factors, including molecular motifs conserved within a microbial species (pathogen-associated molecular patterns or PAMPs), pathogens’ virulence factors (effectors), and subsequent downstream signaling (7). When enabled, NHR can also spark hypersensitive response (HR) – a rapid, localized, and programmed cell death at the point of infection (17).

In addition, plants have constitutive immunity as a part of the NHR: preformed mechanical barriers (leaf topography, waxes, cell wall, volatiles, etc.); various antimicrobial compounds (AMPs) (terpenes, flavonoids, tannins, phytoalexins, phytoanticipins, saponins, etc); and defense proteins, participating in both constitutive and induced immunity (18). Altogether, these processes ensure the inability of a particular pathogen to infect a given host species.

In vertebrates, cancer cells trigger the immune system by analogous mechanisms, such as damage-associated molecular patterns (DAMPs), (19); molecules that may act as “effectors” by suppressing immune cells; metabolic enzymes; and various enzymes eliciting host immune responses (20). They also induce cytotoxicity of the natural killer (NK) cells and cytotoxic T lymphocytes (CTL) prompting immunogenic cell death (ICD), (21) – a concept related to the HR in plants. Like plants, animals produce a wide variety of AMPs and some of them were shown to have anticancer activity (22). Then why all these and other processes associated with multilayered cancer defense, are not capable of creating an environment similar to the non-host resistance in plants?

For that, cancer cells would have to be recognized by an organism as strictly external, “non-self” entities rather than “self” or “altered self” as they are normally perceived, which causes the immune system to stumble. Although cancer cells can still be called “foreign intruders” since they generate neoantigens (23), their identity for the host is not straightforward: they are like a rental apartment a neighbor erected in your own backyard using your building materials.

Besides, unlike plant pathogens which need to penetrate preformed and mechanical barriers, these intruders are of domestic origin and thus often do not have to cope with the first line of organismal defenses but only with the pre-formed genetic defenses and innate immune system.

A sequence of the NHR in plants involves pre-invasive and post-invasive defense responses (18). Pre-invasive responses against cancer in the same manner as they present in plants (physical barriers preventing pathogens from entering the plant) do not occur in animals. Instead, they are a part of the comprehensive (i.e. post-invasive) organismal defenses intended to suppress malignancy at the cellular (e.g. DNA repair, apoptosis, cell-cycle checkpoint, etc), and systemic levels (e.g. immune surveillance). Since this strategy against post-invasive cancer, although robust, ultimately fails, can all the sophisticated organismic responses to cancer assault be rechanneled for it prevention, thus generating a total *non-cancer environment* (NCE), an animal analog of the NHR?

Analogous to NHR in plants—a robust, innate, and broad-spectrum rejection of non-self pathogens — the hypothetical NCE would act as an organism-level defense system that restrains mutated “self” cells, thereby inhibiting tumorigenesis. But if so, would the fundamental biological differences between the two systems make them non-equivalent? Basic molecular and evolutionary processes are conserved in Animalia and Plantae: they are multicellular eukaryotic organisms with similar genetic regulation and developmental processes (24). Furthermore, remarkable similarities between the molecular organization of animal and plant systems for non-self recognition and anti-microbial defense are known, especially evolutionarily conserved molecular mechanisms for innate immunity (25, 26).

Would dramatically enhanced sensitivity of all existing anti-cancer mechanisms create a cancer-free environment? In naked mole-rat (*Heterocephalus glaber*), cells stop dividing due to the hypersensitivity to contact inhibition (27), resulting in a longer life span and suppression of cancer (28). The early contact inhibition is controlled by cyclin-dependent kinase inhibitor p16 in contrast to the regular contact inhibition that is triggered by accumulation of p27, another cyclin-dependent kinase inhibitor (27). Contact inhibition of proliferation is one of the mechanisms of cellular defenses against cancer (29).

Therapeutic upregulation of another inherent anti-cancer pathway, DNA repair, is also feasible by enhancing activity of DNA repair enzymes (30). Modulation of apoptosis, an evolutionary conserved mechanism of cell death (31), toward high apoptotic sensitivity is possible as well. Highly sensitive pro-apoptotic signaling, or priming for apoptosis, is known to occur in developing brain and is responsible for the hypersensitivity of the brain tissue to damage or stress (31). Further, enhancing functional activity of tumor-suppressor protein p53, the “guardian of the genome” (32–34), through regulation of its inhibitors *MDM2* (murine double minute 2 gene) and *MDM4* have been reported (35). Therapeutically increasing the sensitivity of the innate immune system against cancer, for example, by means of enhancing cytotoxicity of the NK is a promising strategy in cancer immunotherapy (36). So is increasing the sensitivity of the adaptive immune system against cancer by multiple approaches, including adoptive cell therapy (37). Tumor microenvironment, a major barrier to effective cancer immunotherapy, can potentially be modulated by combining immune-activating treatments with immune checkpoint inhibitors (5, 38).

Clearly, these measures carry risks of adverse effects, including autoimmunity, tissue degeneration, impaired regeneration, cytotoxicity, and many others. Nevertheless, these potential risks are manageable and can be reduced (39–43).

Thus, considering that many if not all non-immune and immune defenses against cancer can be reinforced and elevated to a higher level, an establishment of the NCE in animals equivalent or comparable to the NHR in plants deems possible. One way to accomplish that would be a combined rather than individual, intensification and continuous preservation of all these processes at the elevated level by virtue of some broad-based elicitor(s) capable of acting on main components of the anti-cancer defenses. Importantly, this would not be a therapeutic treatment of the *existing* malignancy, but instead an initiation of the more preemptive and persistent defensive state of an organism as compared to its ordinary safeguarded status.

This “upgraded” biological condition could be reminiscent of a highly adaptive, “fight or flight” response intended for an immediate survival under acute stress (44). However, unlike the short-term “fight or flight” response, it would have to be a more comprehensive and lasting state constantly supported by all aspects of the innate and adaptive immunity. While chronic cellular stress may promote cancer, it may also suppress it or make it vulnerable (45). Perhaps cognitive, physiological, hormonal, metabolic, and immune mechanisms involved in the sudden stress response along with specific modulations of the anti-cancer processes mentioned above can offer an answer on what integrative catalyst or substance could possibly be utilized to achieve this ameliorated organismal condition of the absolute NCE.

**Figure 1:**
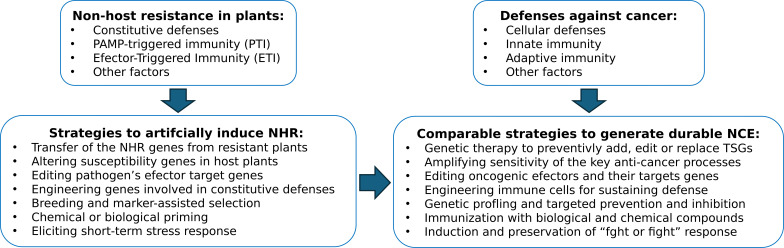
Initiation of the non-cancer environment (NCE) as an analog to the non-host resistance (NHR) in plants. Proposed approaches for generating hypothetical NCE state modeled after NHR in plants. TSGs, tumor suppressor genes.

An alternative or rather broadened way of carrying out this task could be to incorporate approaches used for artificial induction or engineering the NHR in plants (**Figure 1**). The NHR is believed to be a multi-gene trait, and different methodologies currently exist to elucidate its genetic basis and components, including chemical genetics, mutagenesis-aided forward genetics, reverse genetics, exploring near-host variations, and other strategies (8). Importantly, the NHR is transferable from resistant to susceptible plants by transformation methods, genome editing or crossbreeding (46). For example, the expression of non-host genes that are targets of pathogens effectors enhanced disease resistance in host plants: expression of AtPUB33, an E3 ubiquitin ligase and a non-host *Arabidopsis* ortholog of the *Phytophthora infestans* effector target in potato called StUBK, increased resistance to *P. infestans* in two different host species, potato and *Nicotiana benthamiana* (47). Known genetic architectures of cancer suppression such as *BRCA1* and *BRCA2* genes of homologous recombination repair (48); proliferation control genes *TP53* and *RB1* (33, 49); or *APC* gene regulating cell growth (50) can potentially be utilized toward this alternative approach of engineering the NCE. Furthermore, a molecular machinery associated with cancer-hallmarks (CHs) in humans and plants was recently deduced by a comparative genomics method resulting in identification of 2223 homologs of CHs genes in *A. thaliana* (51). Among them were “resisting to cell death” (RCD) genes that in plants are associated with HR: 1072 orthologs were found in *H. sapience* and 609 *in A. thaliana.* Perhaps those RCD orthologs identified in *H. sapience* genome could also be potential candidate genes for engineering NCE.

In addition to genetic engineering, various chemical elicitors and biological priming can be implemented to trigger defense responses in both non-host and host plants (52–54).

Since many genes and processes involved in the cellular and immune responses to oncogenesis are increasingly known, a strategy resembling that used for the induction of the NHR in plants can potentially be applied to generate a durable NCE state in animals. This is particularly relevant given Nature’s existing blueprints that can be used to create NCE: for instance, high-level cancer resistance mechanisms found in some animal species – naked mole-rats, big-footed bats, elephants, and whales (27, 55–57). Interestingly, however, that so called Peto’s paradox proposing association between cancer prevalence and body size (58), and relevant to the latter two species, was recently rejected with new evidence showing that larger species do, in fact, experience higher cancer rates (59).

Whereas NHR exists as a multi-layered form of plant immunity that enacts as a whole almost immediately upon detecting a threat, its artificial induction is likely to be a sequential process in which a single step, for example transfer of the NHR genes from resistant to susceptible plants, could be sufficient to render immunity to a particular pathogen.

The NCE, on the other hand, being not there yet *per se*, is represented by a complex, interconnected but highly vulnerable protective system. It would have to be redesigned from the available organismal resources as a single and ready-to-act operational unit similar to the NHR and possessing a crucial ability to perceive malignancies as “foreign” to the immune system by recognizing cancer-related neoantigens and stress signals (23, 60). This restructuring of the standard defense mechanisms to generate NHR-like comprehensive prevention system will likely require two major steps: i. instant signaling prompted by cancer-recognition events followed by ii. sensitization and upgrade of the existing anti-tumor network.

In line with the NHR, in which the major recognition event is PAMP-triggered immunity (PTI) activated by microbial molecular signatures, cancer-recognition signals initiating quick NCE response must be associated with different biomarkers indicating that transformation of normal cells into malignant is underway (61, 62). It is essential that these cancer fingerprints are of the “non-self” origin and recognized by the immune system as antigens thus stimulating it for the attack (63). Yet, this recognition alone frequently fails because cancers routinely escape immune destruction (64). The escape process, called “immunoediting” (65) strongly reminds the zigzag model illustrating plant response to infection (66).

It consists of three phases, each presenting a distinct barrier subsequently overpowered by cancer cells evading the organism in the end: elimination, equilibrium and escape (64, 65). While eliminating tumors along the way, the immunoediting also plays a dual role of creating cancer phenotypes that escape immune surveillance (65). Likewise, the “zigzag” arrangement of the plant immune system can completely stop pathogen’s colonization (PTI), result in effector-triggered susceptibility (ETS), or in effector-triggered immunity (ETI). The latter can drive pathogens to avoid elimination by gaining new effectors through natural selection (ETS), (66). This phase can circle back to ETI again due to plant regrouping its alleles of resistance (*R*) genes encoding polymorphic nucleotide-binding leucine-rich repeat (NB-LRR) proteins – a direct result of the continuous co-evolution of host *R* genes and pathogen effectors (66).

In contrast, after the third phase of immunoediting (immune escape), the immune system is considered defeated and can no longer block tumors (64, 65). Extending the first phase of immunoediting (elimination of the newly formed tumor) by enhancing activity of immune cells, improved neoantigen presentation, vaccination, oncolytic viruses, etc. - would be a major breakthrough en route to the formation of NCE.

Animals and humans do not have *R*-genes on a par with those in plants. However, they do share the closest resemblances such as pattern recognition receptors (PRRs) of the innate immune response, for example, intracellular NOD-like receptors (NLRs) sensing cancer-derived DAMPs and pathogen-associated PAMPs; membrane-bound Toll-like receptors (TLRs), (67); and transmembrane T-cell Receptors (TCRs) of the adaptive immune system recognizing cancer-related peptides (68). The animal proteins *Apaf-1* (mammals) and *CED-4* (*C. elegans*) also share significant structural and functional homology with plant NB-LLR proteins encoded by most *R* genes (67, 69).

The TCRs can recognize tumor-related neoantigens, which will lead to a clonal proliferation of the specific T cell clones and tumor killing (70), similarly to a formation of new alleles of resistance (R) genes effective against modified pathogen’s effectors. Complete tumor elimination, however, is limited due to the heterogeneity of neoantigens within a single tumor. Consequently, a reversal of immune escape, the same as in the zigzag pattern, does not normally happen and has to be induced therapeutically (71). It is worth noting though that neoantigens have emerged as a primary driver of the immune response, offering an effective way to address the challenges caused by their heterogeneity (72).

In rare cases, spontaneous tumor regression (SR) occurs naturally, without intervention (73). Although SR mechanisms are currently elusive, they bear a resemblance to NHR and mirror the proposed state of NCE by robust activation of all anti-tumor resources.

One of the reported features associated with the SR is coinfection with pathogenic microorganisms promoting a defensive mode of the immune system by stimulation of antigen-presenting cells (APC) (74, 75). The involvement of pathogens in SR response is another hallmark linking it to the NHR and potentially to the establishment of NCE, as infection could be that very signal triggering cancer-recognition events by presentation of cancer biomarkers via APC. Interestingly, although the evidence is almost nonexistent, tumor reversion can also occur in plants infected by a pathogenic bacterium *A*. *tumefaciens*. It was demonstrated in the late 1950s that crown-gall tumor cells could be transformed into benign by series of graftings to healthy plants (76).

A second major step to rearrange standard defense mechanisms against cancer presumably requires sensitization and upgrade of the existing anti-tumor network. As evident from the research on contact inhibition, therapeutic upregulation, priming, and enhancement of sensitivity of cellular and immune responses (27, 29, 31, 35, 36, 37, 71), as well as from the studies on SR (75, 77), this task is feasible. Moreover, as all organismal resources and arrays of defense mechanisms against cancer are readily available and highly modifiable, their recurrent, preventive, and stress-induced upgrade would deem natural. Just like an upgrade of an existing version of a computer program by running scripts and commands to change its configuration.

As noted earlier, one example of the command to step up and maximize could come from cancer-associated, presumably non-carcinogenic, microbial infections by way of DAMPs and activated APCs. Antitumor efficacy can also be augmented by beneficial microbes: a recent study showed that a new strain of the bacterial genus *Hominenteromicrobium*, a probiotic member of the human gut microbiome, activated tumor-specific CD8+ T cells, enhancing antitumor responses in mice (78). Hormonal activation, signals inducting apoptosis, ischemia, or epigenetic changes can also contribute to the upgrade of anti-tumor network (79). The more precise mechanisms would likely stem from deciphering the SR response (80).

After signals to launch NCE-like response are initiated and the status is implemented through optimization of anti-cancer defenses, further actions will be necessary to make the process sustainable and, more importantly, reproducible. So that any uncontrollably proliferating cells will be treated by an organism as non-adapted invaders and the ability to enhance the body’s anti-cancer defenses will be retained in the future. Plant immune system can be primed to generate faster, stronger, and long-lasting defense response upon pathogen’s attack (81). Defense priming in plants can be triggered not only by pathogens, but also by beneficial microorganisms, alterations in primary metabolism, synthetic and chemical compounds (81, 82). Formerly called sensitization (83), defense priming involves amplification of extracellular stimuli by mitogen-activated protein kinases (MPKs); chromatin modifications; systemic acquired resistance (SAR); molecular modifications of primary metabolism; rapid accumulation of reactive oxygen species (ROS); and other signaling processes and pathways (81–83). Defense priming, as a form of induced resistance, can also contribute to the NHR.

Against this plant background, a fairly new concept of trained immunity, an enhanced immunity in vertebrates that follows initial exposure to a pathogen (84, 85), may be potentially viewed as a process able to provide consistency to the NCE. The idea of trained immunity in vertebrates initially came from similarities with long known SAR phenomenon in plants, when plants are broadly and systemically protected for extended periods of time after initial local infection (84). One of the key characteristics of trained immunity is a broad and rapid response to PAMPs and DAMPs leading to reprogramming of different cell types of the innate immune system and heightened state of immune cell effectors (85). Same as in plants, trained immunity in cancer can be artificially induced by therapeutic agents like β-glucan or Bacillus Calmette-Guerin (BCG) vaccination, or happen naturally after infections, for example, with influenza A virus or hepatitis B virus (85).

**Figure 2:**
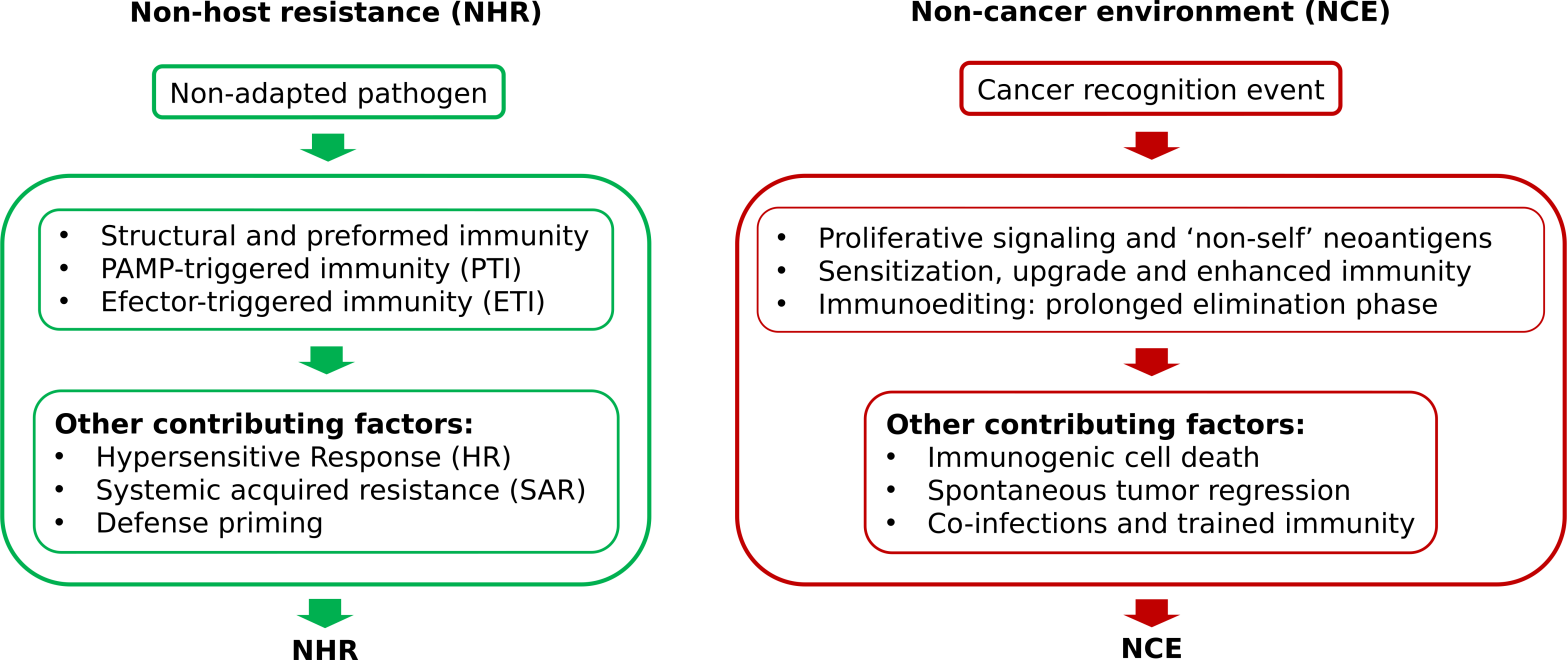
Conditions essential for unveiling non-host resistance (NHR) and hypothetical non-cancer environment (NCE) states.

Summarizing, potential steps or conditions imperative for unveiling the NCE state include initial proliferating signaling followed by instant sensitization, upgrade and preservation of the continuing elevated status of all available anti-cancer resources. In a general way, those are the same requirements needed for the establishment of NHR in plants (**Figure 2**). Since the NHR trait is inherited, it may well be that NCE state is intrinsic likewise and only needs to be “uncaged” from idleness by yet unidentified but readily available factor(s).

The NHR in plants has been known for many years and experimentally tested (7); each of the potential components and manifestations that may prompt and accompany the NCE state (sensitization, priming, vaccines, ICD, SR, elimination phase of immunoediting, etc.) has been studied and empirically verified as well. It is therefore possible, based on the available data, to develop exploratory *in vitro* and *in vivo* models simulating NCE conditions and their effect on the process of tumorigenesis.

## Conclusions

While individual enigmas pertaining hypothetical establishment of the NCE state and even the order of the proposed events could be learned from the NHR and mechanisms of plant immunity in general, linking them together into the closely tied, vigilant, and sensitized protective network is a challenging task. Nevertheless, both the concept and the obvious possibility of the natural occurrence of the NCE appear strongly plausible, especially keeping in mind the resilience and efficiency of the comparable NHR process in plants and striking similarities in the molecular mode of plant and animal immune systems (25). Moreover, within the current accumulated knowledge of the processes related to the NHR and to the body’s natural and induced defenses against cancer, this hypothesis appears to be experimentally testable. The testing could be approached in multiple ways including genetically engineering animals with traits that are thought to confer cancer resistance or enhancing their immune response by a combination of immunotherapies and trained immunity (**Figure 1**).
